# Selenium Bio-Nanocomposite Based on *Alteromonas macleodii* Mo169 Exopolysaccharide: Synthesis, Characterization, and In Vitro Antioxidant Activity

**DOI:** 10.3390/bioengineering10020193

**Published:** 2023-02-02

**Authors:** Patrícia Concórdio-Reis, Ana Catarina Macedo, Martim Cardeira, Xavier Moppert, Jean Guézennec, Chantal Sevrin, Christian Grandfils, Ana Teresa Serra, Filomena Freitas

**Affiliations:** 1 Associate Laboratory i4HB—Institute for Health and Bioeconomy, School of Science and Technology, NOVA University Lisbon, 2829-516 Caparica, Portugal; 2 UCIBIO—Applied Molecular Biosciences Unit, Department of Chemistry, School of Science and Technology, NOVA University Lisbon, 2829-516 Caparica, Portugal; 3 iBET, Instituto de Biologia Experimental e Tecnológica, Apartado 12, 2781-901 Oeiras, Portugal; 4 Instituto de Tecnologia Química e Biológica António Xavier, Universidade Nova de Lisboa (ITQB NOVA), Av. da República, 2780-157 Oeiras, Portugal; 5 Pacific Biotech BP 140 289, Arue Tahiti 98 701, French Polynesia; 6 AiMB (Advices in Marine Biotechnology), 17 Rue d’Ouessant, 29280 Plouzané, France; 7 Interfaculty Research Centre of Biomaterials (CEIB), University of Liège, B-4000 Liège, Belgium

**Keywords:** *Alteromonas macleodii* Mo 169, exopolysaccharide (EPS), selenium nanoparticles (SeNP), bioactive material, blue biotechnology

## Abstract

In this study, the novel exopolysaccharide (EPS) produced by the marine bacterium *Alteromonas macleodii* Mo 169 was used as a stabilizer and capping agent in the preparation of selenium nanoparticles (SeNPs). The synthesized nanoparticles were well dispersed and spherical with an average particle size of 32 nm. The cytotoxicity of the EPS and the EPS/SeNPs bio-nanocomposite was investigated on human keratinocyte (HaCaT) and fibroblast (CCD-1079Sk) cell lines. No cytotoxicity was found for the EPS alone for concentrations up to 1 g L^−1^. A cytotoxic effect was only noticed for the bio-nanocomposite at the highest concentrations tested (0.5 and 1 g L^−1^). In vitro experiments demonstrated that non-cytotoxic concentrations of the EPS/SeNPs bio-nanocomposite had a significant cellular antioxidant effect on the HaCaT cell line by reducing ROS levels up to 33.8%. These findings demonstrated that the *A. macleodii* Mo 169 EPS can be efficiently used as a stabilizer and surface coating to produce a SeNP-based bio-nanocomposite with improved antioxidant activity.

## 1. Introduction

Selenium (Se) is an essential trace mineral for biological functions, including the synthesis of selenoenzymes capable of regulating important physiological functions of the human body [1]. Se is present in the selenocysteine residue (SEC) of various antioxidant enzymes (e.g., glutathione peroxidase (GPX), thioredoxin reductase (TXNRD), and selenoprotein P(SELENOP)), and acts as their redox center, being essential for their activity [2]. These antioxidant enzymes can effectively protect the membrane structures of organisms from oxidative damage [3]. Apart from its antioxidant activity, Se intake has several health benefits, including the regulation of the thyroid hormone metabolism, an anticancer effect (mainly chemopreventive), and the modulation of the immune system response in inflammatory disorders (e.g., diabetes, bone toxicity, colitis, and drug-induced toxicity) [1,2]. Nonetheless, Se’s biological activity strongly depends on its chemical form and the absorbed dose [4]. With most European agricultural soils being classified as having low Se concentrations [5], sodium selenite (Na_2_SeO_3_), sodium selenate (Na_2_SeO_4_), or selenomethionine (SeMet) are commonly found as Se sources in food supplements [4]. However, inorganic Se and most organic Se compounds can be toxic at higher concentrations; thus, there is a very narrow therapeutic window between effective beneficial doses and toxic levels [3,4]. Therefore, there is a need for new Se compounds that are biocompatible with efficient bioactivities. In response to this issue, nanosized selenium particles (SeNPs) have been explored since they can dramatically reduce the toxicological concerns associated with Se [2,6]. Additionally, SeNPs exhibited improved bioavailability but also pharmacological activities with anticancer, anti-microbial, and antioxidant capacities [1,2,7]. Nonetheless, their clinical applications have some limitations since SeNPs are unstable and tend to aggregate and precipitate [1,7,8]. Another major drawback of SeNPs’ application is their poor cellular intake [2].

Natural polysaccharides have emerged as an attractive solution to solve these problems [1,9]. In addition to being considered generally safe, these high molecular weight polymers can be used as soft template regulators to control crystal nucleation and growth during SeNPs’ synthesis, resulting in nanoparticles with an ideal particle size and morphology [1,3]. Additionally, the conjugation of SeNPs with polysaccharides improves the efficiency of the interaction between the SeNPs and the target cells, since protein adsorption is prevented by the polysaccharides’ hydrophilic groups [1]. Several studies have demonstrated the therapeutic potential of SeNPs coated with natural polysaccharides. For instance, SeNPs decorated with the exopolysaccharide produced by *Cordyceps sinensis* exhibited a protective effect against oxidative stress in HepG2 cells by increasing cell viability, restoring cell and nucleus morphology, and reducing lactate dehydrogenase levels [7]. Another example are the *Grateloupia Livida* polysaccharide-functionalized SeNPs, which demonstrated significant scavenging capacity, low oral acute toxicity, and selective cytotoxicity towards various human cancer cell lines but not towards normal cells [10]. Additionally, the SeNPs coated with *Gracilaria lemaneiformis* polysaccharide showed an anti-glioblastoma activity superior to that of the NPs alone. The *G. lemaneiformis* polysaccharide improved the SeNPs’ cellular uptake and the specific binding to the αv-β3 integrin receptors overexpressed on U87 glioma cells [11].

Characterized by unique ecological features, marine environments are a rich source of novel polysaccharides with unusual chemical compositions and structural characteristics, such as those found in the exopolysaccharides (EPS) produced by marine bacteria [12,13]. These EPS of marine origin often exhibit interesting properties, including excellent biocompatibility and biodegradability, anti-microbial, immunomodulatory, antioxidant, and wound-healing abilities, and the capacity to form structures such as hydrogels and films [14,15]. Thus, the EPS produced by marine bacteria might be of value for the development of therapeutic products based on SeNPs.

Recently, the EPS produced by the marine bacterium *Alteromonas macleodii* Mo169, composed of two high molecular weight fractions (1.6 and 4.6 MDa), was characterized and investigated as a biomaterial for biotechnological applications [15]. The EPS had a high anionic character as it was mainly composed of glucuronic acid (GlcA, 39.3 mol%), with a minor content in mannose (Man, 12.8 mol%), glucose (Glc, 11.2 mol%), galacturonic acid (GalA, 10.4 mol%), galactose (Gal, 4.0 mol%), and glucosamine (GlcN, 2.4 mol%) [16]. Acyl substituents, namely sulfate (5.3 wt.%), lactate (0.63 wt.%), and pyruvate (0.063 wt.%), were also detected in the EPS’ composition [16]. GlcA, Man, Glc, and Gal were present as (1 → 4)-linked residues, with ramifications or substitutions at positions 2 and/or 3 [16]. Its capacity to form films and gels along with its interesting rheological properties suggested that the EPS might be a suitable thickening or structuring agent in tissue engineering, wound management, or drug delivery applications [15,16]. Based on its high anionic character and high viscosity, *A. macleodii* Mo 169 EPS might have the potential to be used as a stabilizer and dispersing agent in the preparation of stable and uniform SeNPs [8], which was assessed in this study. The size and morphology of the obtained SeNPs were evaluated and the EPS/SeNPs bio-nanocomposite was characterized in terms of their morphology, size, zeta potential, and selenium concentration. Besides, the biocompatibility and antioxidant ability of the synthesized bio-nanocomposite were investigated through in vitro assays.

## 2. Materials and Methods

### 2.1. Materials

The EPS was produced by *Alteromonas macleodii* Mo 169 (CNCM I-5374) in a 1 L fermenter, as previously described [15]. The EPS was recovered by high-speed centrifugation (20,000× *g*, 2 h, 25 °C) and purified by ultrafiltration, concentrated, and lyophilized [15]. Sodium selenite (Na_2_SeO_3_) (≥95.0%) and ascorbic acid were obtained from Sigma-Aldrich(Dorset, UK).

### 2.2. Preparation of the Bio-Nanocomposites

The SeNPs were synthesized according to the procedure described by Yan et al. [17], with minor modifications. A 2 g L^−1^ EPS solution was prepared (5 mL), and the pH was adjusted to ~8 with NaOH (0.1 M). Control samples were prepared using deionized water instead of the EPS solution. A quantity of 500 µL of a 100 mM Na_2_SeO_3_ stock solution was added to the EPS or control solutions, to achieve a Se^4+^ concentration of 10 mM. After stirring, 1 mL of an ascorbic acid solution (200 mM) was added to the tubes, followed by incubation at room temperature, for 1 h, protected from the light. SeNPs’ formation was noticed by the appearance of a red color, which was confirmed by UV–visible spectra measurements (CamSpec M509T spectrophotometer) in the wavelength range of 200–800 nm. After NPs’ synthesis, the EPS/NPs bio-nanocomposites were dialyzed using a 12 kDa MWCO (molecular weight cut-off) membrane (ZelluTrans/Roth) against deionized water, for 48 h. The purified bio-nanocomposite was maintained at 4 °C or lyophilized for further analysis. The reactions were performed in triplicates.

### 2.3. Characterization of the Bio-Nanocomposites

The selenium content in the bio-nanocomposite was determined by Inductively Coupled Plasma–Atomic Emission Spectroscopy (ICP-AES) (Ultima, Horiba Jobin-Yvon, France, equipped with a 40.68 MHz RF generator, Czerny–Turner monochromator with 1.00 m (sequential) and autosampler AS500). The zeta potential measurements were performed in a Zetasiser Nano ZS, model ZEN (Malvern Panalytical), adopting electrophoretic cells (disposable folded hair cells; reference DTS1070) at 25 °C. Zeta potential was calculated by adopting the Smoluchowski equation [18]. The particle size of the EPS/SeNPs bio-nanocomposite (diluted 10 times) was determined by dynamic light scattering (DLS) using Photocor equipment (helium–neon laser, 633 nm, 20 mW, 90 °C) and a Brookhaven BI9000 autocorrelator. All measurements were performed at least three times and the raw data were analyzed with Dynals Software (SoftScientific, Tallinn, Estonia). Transmission electron microscopy (TEM) (JEM 1400; JEOL Europe, Zaventem, Belgium) was employed for the determination of the SeNPs’ morphology and size distribution, as described by Concórdio-Reis et al. [18]. Fourier transform infrared (FT-IR) spectra of the samples were recorded on a Perkin-Elmer Spectrum II spectrometer (Waltham, MA, USA) over 500–4500 cm^−1^ after 10 scans. X-ray diffraction (XRD) analysis was performed using a benchtop MiniFlex II X-ray diffractometer from Rigaku (Tokyo, Japan) with a Cu X-ray tube (30 KV/15 mA). The 2ϴ scans were performed from 10° to 60°, with a step size of 0.01°. Thermogravimetric (TG) analysis was conducted using a Thermogravimetric Analyzer Labsys EVO (Setaram, France), with a heating rate of 10 °C min^−1^, from 25 to 500 °C.

### 2.4. Biological Assays

#### 2.4.1. Cell Cultures

Human immortalized keratinocyte cell line HaCaT, obtained from Deutsches Krebsforschungszentrum (DFKZ, Heidelberg, Germany), and the human fibroblast cell line CCD-1079Sk obtained from American Type Culture Collection (ATCC, Manassas, VA, USA) were used in this study. HaCaT cells were cultured in Dulbecco’s Modified Eagle medium (DMEM) supplemented with 10% (*v*/*v*) of heat-inactivated fetal bovine serum (FBS) and 1% (*v*/*v*) penicillin-streptomycin (PS). CCD-1079Sk cells were cultured in DMEM medium supplemented with 10% (*v*/*v*) FBS and 1% (*v*/*v*) non-essential amino acids (NEAA). Cells were maintained at 37 °C with 5% CO_2_, as described by Concórdio-Reis et al. [18].

#### 2.4.2. Cytotoxicity Assays

The HaCaT and CCD-1079Sk cells were seeded into 96-well plates at a density of 4.5 × 10^5^ cells mL^−1^ and 1.5 × 10^5^ cells mL^−1^ and allowed to grow for 72 and 24 h, respectively. Then, the cells were incubated with the EPS and the EPS/SeNPs bio-nanocomposite diluted in the respective culture medium with 0.5% FBS (125–1000 mg L^−1^). Control experiments were performed by incubating the cells with only a culture medium (0.5% FBS). After 24 h, the cells were washed once with PBS (Sigma-Aldrich, St. Louis, MO, USA) and the cell viability was assessed through the CellTiter 96^®^ AQueous One Solution Cell Proliferation Assay (Promega, Madison, WI, USA), which contained MTS reagent. The optical density was measured at 490 nm using a BioTek EPOCH2 Microplate Reader (BioTek Instruments, Winooski, VT, USA) and cell viability was expressed in terms of the percentage of living cells relatively to the control.

#### 2.4.3. Cellular Antioxidant Activity

Cellular antioxidant assays were performed following previously described methods [19,20], with some modifications. HaCaT cells were seeded at a density of 1.4 × 10^5^ cells cm^−2^ in 96 well plates and the formation of intracellular reactive oxygen species (ROS) was monitored using 2′,7′-dichlorofluorescein diacetate (DCFH-DA) as a fluorescent probe. Then, 72 h after seeding, cells were washed with PBS and treated with selected non-toxic concentrations (62.5 µg mL^−1^; 125 µg mL^−1^; 250 µg mL^−1^) of the samples and 25 µM DCFH-DA in PBS for 1 h. Subsequently, cells were washed again with PBS and incubated with the stress inducer (600 µM AAPH in PBS) for 1 h. After that, fluorescence was measured in a FL800 microplate fluorescence reader (Bio-Tek Instruments, Winooski, VT, USA) (Ex/Em 485 ± 20/528 ± 20 nm). The results were expressed as ROS percentage relative to the untreated control (cells treated with DCFH-DA and AAPH). Three independent experiments were performed, each sample was measured in triplicate.

### 2.5. Data Analysis

Three independent experiments were performed in at least duplicate and the results were expressed in terms of mean ± SD (standard deviation). When homogeneous variance and a normal distribution of the data were verified, the results were analyzed by one-way analysis of variance (ANOVA), followed by the Tukey or Bonferroni tests for multiple comparisons. If the data were not normally distributed or in the case of heterogeneous variances, the statistical analysis was performed by Student’s *t*-test. Differences resulting in *p* < 0.05 were considered statistically significant. Adopting Lucia G Software, version 4.80 (Laboratory Imaging s.r.o–Nicon, Praha, Czech Republic), the equivalent diameter of at least 1000 particles was analyzed in view to determine particle size distribution, mean and corresponding percentiles: 10, 50, 90, and 99.

## 3. Results and Discussion

### 3.1. NPs Synthesis

**Figure 1:**
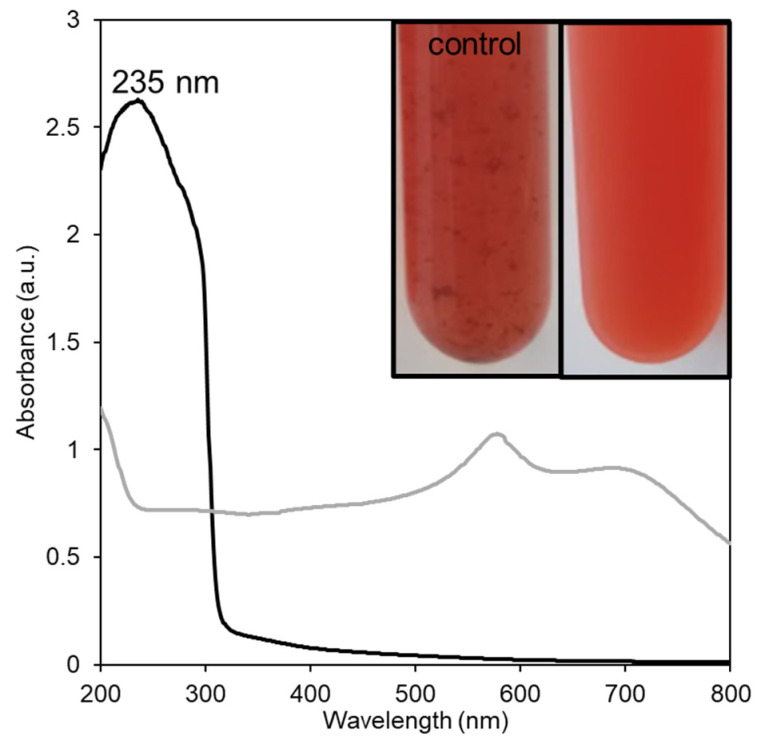
UV-vis absorption spectra and digital photography (insert) of the control (grey line) and the SeNPs bio-nanocomposite prepared with the *A. macleodii* Mo 169 EPS (black line).

For SeNPs’ synthesis, the EPS was investigated for the stabilization of the SeNPs, and ascorbic acid was added to the mixture as a reducing agent. Ascorbic acid was previously used as a reducing agent in the synthesis of SeNPs stabilized by *C. sinensis* fungus Cs-HK1 EPS [8], carboxylated curdlans [17], β-D-glucan [21], chitosan [22], arabinogalactans [9], lectinan [23], gum Arabic [24], pectin [3], and by the polysaccharides of *G. lemaneiformis* [4], *G. Livida* [10], *Oudemansiella raphanipies* [6], *Lycium barbarum* [25], and *Catathelasma ventricosum* [26]. Within 1 h of incubation at room temperature with 10 mM of Se^4+^, the solution’s color changed from colorless to light yellow and, finally, to red (Figure 1, insert), indicating the formation of amorphous or monoclinic SeNPs [8,9,17,25]. This alteration in color may be ascribed to the excitation of surface plasmon resonance (SPR) of SeNP, resulting in the SPR band observed in the UV-vis spectra of SeNPs (235 nm) (Figure 1). The SeNPs synthesized by other polysaccharides presented a SPR band at a slightly higher wavelength (260–273 nm) [3,4,17,21,25]. Nonetheless, differences in the absorption peak were reported when the synthesis conditions were altered [8,9] and might be related to differences in SeNPs’ crystallinity and particle size [9]. For instance, in the study that reported the use of arabinogalactan as a stabilizer for SeNPs’ synthesis, an increase in SeO_3_^2−^ concentration from 30 to 120 mM resulted in particles with higher dimensions, as well as an increase in the intensity of the absorption band, and a shift in its position from 260 nm to higher wavelengths was noticed [9]. Thus, it is expected that lower selenium concentrations, such as the 10 mM used in this study, would result in SPR bands located at lower wavelengths. Additionally, the study performed by Xiao et al. [8] using *C. sinensis* Cs-HK1 EPS for the stabilization of the SeNPs showed that the ratio of selenium to EPS can also affect the characteristics of the SPR band, including its intensity and width. An increase in the Se: EPS ratio from 1:1 to 1:3 led to the formation of unstable large-sized SeNPs, characterized by a broader SPR band with a peak at a higher wavelength (306 nm) [8].

Although the control experiment without the addition of EPS also changed from colorless to red, the presence of precipitates was noticed (Figure 1, insert). Similarly, previous studies reported the formation of brown/black aggregates that precipitated after Se^4+^ incubation with ascorbic acid in the absence of the stabilizer agent [8,9,17,25]. Bare SeNPs are known to be highly unstable in aqueous solutions. Due to their high surface energy, these NPs tend to form strong interparticle bonds that result in their aggregation and precipitation [8,17,25]. Thus, adding natural polysaccharides, such as *A. macleodii* Mo 169 EPS, is essential for the synthesis of stable and well-dispersed SeNPs. In the presence of the EPS, NPs’ synthesis occurred in the polysaccharide’s molecular microenvironment and the EPS acts as a soft template to stabilize the SeNPs by controlling the crystal nucleation and growth processes [1]. Moreover, the functional groups of the polysaccharide would facilitate the interaction of the SeNPs with the target cells and their lipidic barriers [1].

### 3.2. NPs Characterization

#### 3.2.1. Composition

**Figure 2:**
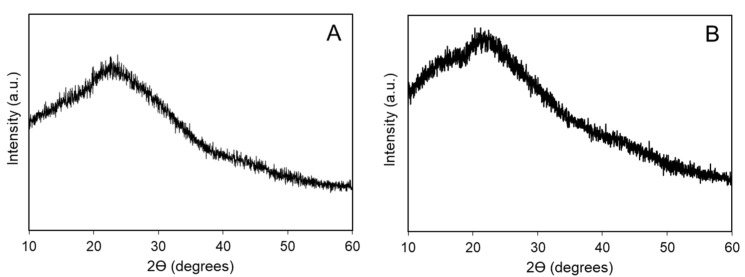
Diffractograms of *A. macleodii* Mo 169 EPS (**A**) and its EPS/SeNPs bio-nanocomposite (**B**).

After purification by dialysis, the EPS/SeNPs bio-nanocomposite contained 183.8 mg L^−1^ of selenium. Nonetheless, the diffractogram of the bio-nanocomposite did not present the typical Bragg peaks of crystalline selenium (Figure 2B). Only one large band at about 20° was detected in both diffractograms (Figure 2), which can be attributed to the polysaccharide’s amorphous nature. Similar results were observed in previous reports [3,4,6,10,17,21,23]. Although the typical peaks of crystalline Se appear in the XRD spectrum of the purified SeNPs, these disappeared in the presence of the polysaccharides *C. sinensis* Cs-HK1 EPS [8] and carboxylated curdlan [17], and the bio-nanocomposites showed the same pattern as the amorphous polysaccharide, suggesting that the SeNPs were bound by the polysaccharide matrix, leading to the occurrence of amorphous SeNPs [8,17].

#### 3.2.2. Colloidal Stability

The colloidal stability of the synthesized EPS/SeNPs bio-nanocomposite was evaluated through the measurement of its zeta potential. The EPS/SeNPs bio-nanocomposite presented a high negative zeta potential of −46.43 ± 1.36 mV. This high electrokinetic potential can easily explain this enhancement of the physicochemical stability of the colloidal suspension through ionic, or electrostatic, repulsion afforded by the anionic charges of EPS which has migrated on their surface [18,27]. This value was consistent with the values found in the literature for polysaccharide-stabilized SeNP, namely the polysaccharides from *G. lemaneiformis* (−47.1 mV) [4] and *G. Livida* (−46.77 mV) [10]. Smaller negative potential values were reported for SeNPs prepared with carboxylated curdlans (−17 to −28 mV) [17], *O. raphanipies* polysaccharide (−14.1 mV) [6], and *L. barbarum* polysaccharides (−37 mV) [25], which might be related to differences in composition (e.g., content in uronic acids) and molecular weight between polysaccharides [17,25].

#### 3.2.3. SeNPs Morphology

**Figure 3:**
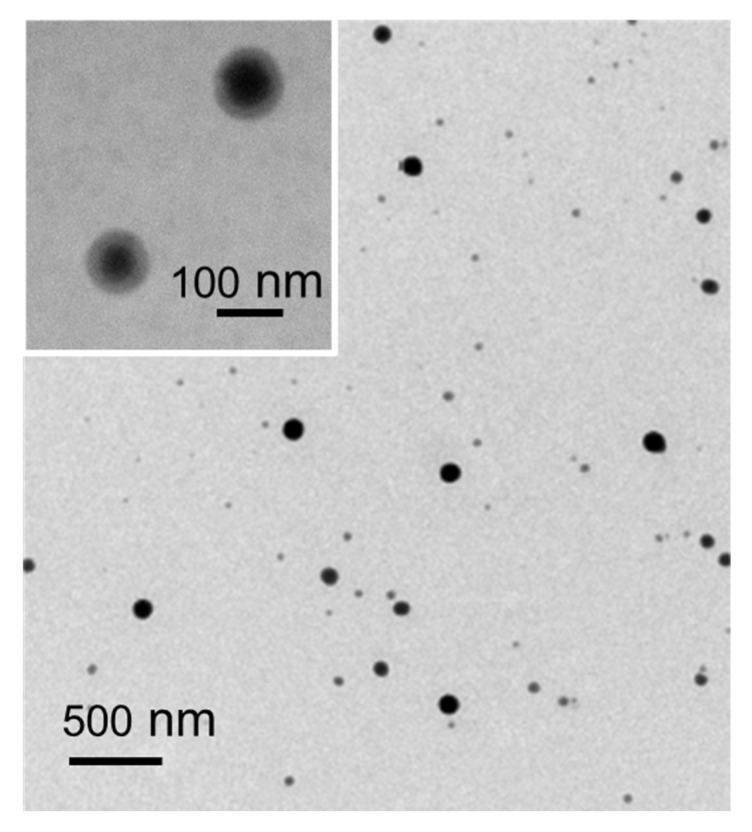
TEM images of the bio-nanocomposite suspensions prepared with SeNPs stabilized by *A. macleodii* Mo169 EPS.

TEM micrographs of the purified EPS/SeNPs bio-nanocomposite showed that the synthesized SeNPs were monodispersed and homogenous spherical particles (Figure 3). Interestingly, a fainted sphere-like layer around the SeNPs was observed (Figure 3, insert). This coating layer can be assigned to the EPS, suggesting that the high molecular weight polysaccharide had an important role in the dispersion and stabilization of the SeNPs. No SeNPs’ aggregates were noticed in the TEM images (Figure 3), which occurred in the previous study where carboxyl curdlan Cur-4 was used as a stabilizer and capping agent [17]. In that study, the authors concluded that the compact random coils that Cur-4 exhibited might have limited the interaction between the polymer’s chains and the SeNPs, envisaging the importance of chain conformation on SeNPs’ stability, size, and morphology [17].

#### 3.2.4. SeNPs and Bio-Nanocomposite’s Size

**Figure 4:**
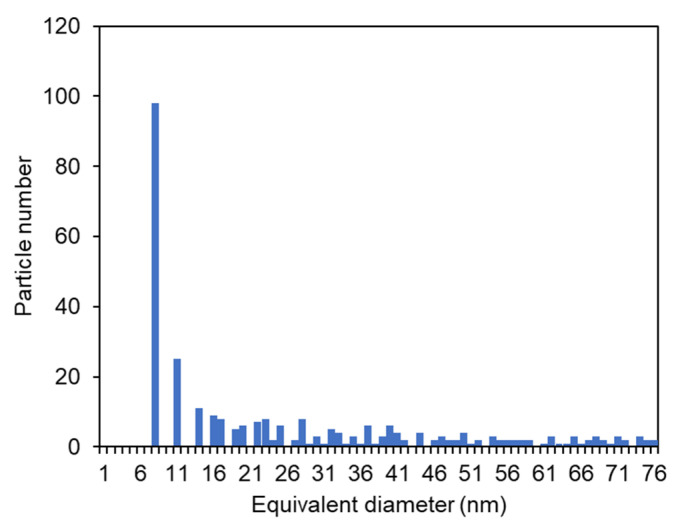
Particle size distribution of the SeNPs prepared with *A. macleodii* Mo 169 EPS.

The particle size distribution of the SeNPs was homogenous, ranging from 22 to 76 nm for percentile 50 and 90, respectively (Figure 4). With an average particle size of 32 nm, the SeNPs’ size is within the range of values reported in the literature (Table 1). SeNPs with sizes ranging from 5 to 200 nm have shown improved protective properties in vitro and in vivo against the oxidative effects of free radicals [28,29], which indicated the potential antioxidant activity of the EPS/SeNPs bio-nanocomposite.

The mean hydrodynamic diameter of the colloidal SeNPs coated by the EPS was determined by DLS (Table 1). The EPS/SeNPs bio-nanocomposite had a diameter of 297 ± 4 nm, a value significantly higher in comparison with that obtained in TEM (22–76 nm). Nonetheless, this difference in particle size was also previously reported for other EPS/NPs bio-nanocomposites [4,8,10,30,31,32,33,34], and can be attributed to the differences in both techniques (e.g., detection method or sample conditioning) [30]. Additionally, the hydrodynamic diameter includes the hydration layer and polymer coating, thus resulting in larger values [10,17,34].

Nonetheless, in this study, the SeNPs stabilized by *A. macleodii* Mo 169 EPS presented a higher value (297 ± 4 nm) compared with those reported in the literature (Table 1). As examples, those prepared with the galactose-rich polysaccharides of *G. lemaneiformis* (92.5–137.7 nm) [4] and *G. Livida* (115.4 nm) [10], *Larix principis-rupprechtii* arabinogalactans (94.24–173.2 nm) [9], lectinan (100 nm) [23], gum Arabic (145–170 nm) [24], and the *L. barbarum* polysaccharide composed of arabinose, xylose, glucose, and galactose (105.4 nm) [25]. Interestingly, all these polysaccharides presented a lower Mw (0.092–18.4 kDa) than that found for *A. macleodii* Mo 169 EPS (1.6 and 4.6 MDa) [15]. The closest value was found for SeNPs prepared with carboxylated curdlan Cur-4 with a Mw of 0.57 MDa (243.4 nm) [17], suggesting that the large Mw of *A. macleodii* Mo 169 EPS could cause the larger particle size of the EPS/SeNPs bio-nanocomposite.

#### 3.2.5. EPS-NPs Interaction

**Figure 5:**
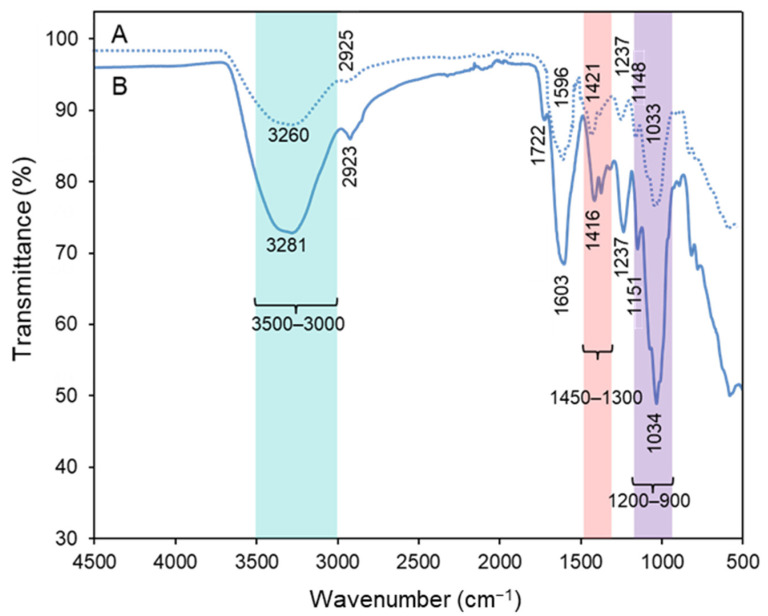
FTIR spectra of purified *A. macleodii* Mo169 EPS (**A**) and its SeNPs bio-nanocomposite (**B**).

FTIR analysis was carried out to investigate the surface functional groups of the *A. macleodii* Mo169 EPS involved in the stabilization of the SeNPs. An alteration in the shape and intensity of the band found between 3000 and 3500 cm^−1^, which corresponds to the stretching frequencies of hydroxyl groups (O-H) [9,15,32], was found in the bio-nanocomposite in comparison with the EPS alone (Figure 5). A shift to a lower wavenumber was noticed in the peak of the stretching vibration of C-H that appeared at 2925 cm^−1^ in the EPS (Figure 5A) [9,17]. Significant alterations were found in the adsorption region characteristic of the C=O asymmetric (1596 cm^−1^) and symmetric (1300–1450 cm^−1^) stretching vibrations of the carboxylates from the uronic acids [15,35], which suggests that these groups played an important role in the stabilization of the SeNPs. Additionally, alterations were noticed in the region of 1722 cm^−1^ assigned to the C=O stretching of the acyl substituents [18]. These results revealed the importance of hydroxyl and carboxylate groups in the stabilization of the SeNPs. In previous studies, SeNPs’ aggregation seemed to be avoided through the interactions between the NPs and the hydroxyl groups of different polysaccharides, including lectinan [23], arabinogalactan [9], pectin [3], gum Arabic [24], curdlan [17], and *C. sinensis* EPS [8], and *L. barbarum* [25], and *Lignosus rhinocerotis* [21] polysaccharides. In addition to the hydroxyl groups, the imino groups of *G. Livida* and *G. lemaneiformis* polysaccharides also seemed to be involved in SeNPs’ stabilization [4,10].

### 3.3. Biological Assays

#### 3.3.1. Assessment of Cytotoxicity

**Figure 6:**
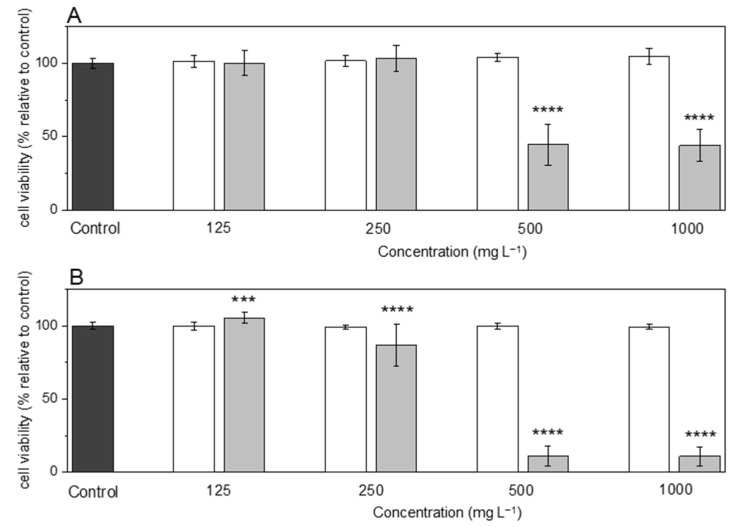
Cytotoxicity studies of *A. macleodii* Mo 169 EPS (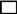) and the EPS/SeNPs bio-nanocomposite (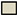) on CCD-10795k (**A**) and HaCaT (**B**) cell lines after 24 h incubation. Control experiments (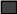) were performed by incubating the cells with only culture medium. Statistically significant differences comparing samples with the control were calculated according to the *t*-test (***, *p* ≤ 0.001, **** *p* ≤ 0.0001).

The EPS and EPS/SeNPs bio-nanocomposite biocompatibility was investigated on human skin cell lines, namely CCD-1079Sk fibroblasts (Figure 6A) and HaCaT keratinocytes (Figure 6B). As described in ISO 10993-5, a cytotoxic effect was considered when cell viability decreased below 70%. As presented in Figure 6, the EPS alone did not show any cytotoxic effect on either cell line for concentrations up to 1000 mg L^−1^, indicating its biocompatibility. Regarding the cytotoxicity of the EPS/SeNPs bio-nanocomposite, concentrations of 500 mg L^−1^ (containing 47 mg_Se_ L^−1^) and 1000 mg L^−1^ (containing 92 mg_Se_ L^−1^) were cytotoxic, causing a reduction in CCD-1079Sk and HaCaT cell viability superior to 55% (Figure 6A) and 89% (Figure 6B), respectively. Nonetheless, in the presence of 125 mg L^−1^ and 250 mg L^−1^ of EPS/SeNPs bio-nanocomposite (11 mg_Se_ L^−1^ and 23 mg_Se_ L^−1^, respectively), no cytotoxic effect was considered since cell viability of both cell lines exceeded 87 ± 15% (Figure 6). Similar results were obtained by other authors for SeNPs (83.6 nm) coated with *G. lemaneiformis* polysaccharides, where RAW 264.7 cells’ viability was maintained above 77% for concentrations up to 20 mg_Se_ L^−1^ [4]. Moreover, in another study, RWPE-1 cells maintained their viability in the presence of 400 mg L^−1^ of pectin-coated SeNPs [3], a concentration superior to that found in this study (250 mg L^−1^). In addition to the differences in cell lines’ sensitivity towards metals [18], the size of those SeNPs was superior (41 nm, compared with 32 nm for *A. macleodii* Mo 169 EPS), and the content in Se of the bio-nanocomposite might also be different, resulting in the differences observed.

#### 3.3.2. Evaluation of Cellular Antioxidant Activity

**Figure 7:**
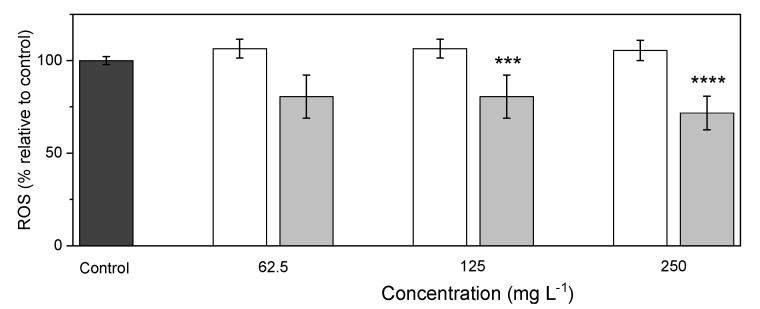
Antioxidant assay. Effect of *A. macleodii* Mo169 EPS (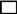), and the EPS/SeNPs bio-nanocomposite (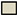) on the inhibition of AAPH-induced ROS production in HaCaT cells. Control experiments (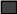) were performed by incubating the cells only with AAPH. Statistically significant differences comparing samples with the negative control were calculated according to the *t*-test (***, *p* ≤ 0.001, **** *p* ≤ 0.0001).

Free radicals such as reactive oxygen species (ROS) can cause oxidative stress in essential cellular structures of living organisms, leading to altered functionality [19,36]. Many oxidative stress-related diseases, such as cardiovascular and immunity diseases, type-II diabetes, cancer, or aging, are related to the accumulation of excessive ROS [7]. Antioxidants, by reducing the level of ROS, can be effective in the protection of the cells against oxidative stress [7]. The capacity of the *A. macleodii* Mo 169 EPS and its SeNPs bio-nanocomposite to reduce AAPH-induced ROS production in HaCaT cells was evaluated (Figure 7). The EPS alone did not reduce ROS generation at a cellular level. Nonetheless, EPS/SeNPs bio-nanocomposite concentrations of 125 mg L^−1^ (11 mg_Se_ L^−1^) and 250 mg L^−1^ (23 mg_Se_ L^−1^) significantly reduced ROS production (*p* ≤ 0.001) by 25.9% and 33.8% (Figure 7), suggesting a dose-dependent cellular antioxidant capacity. Comparable results were reported in the literature for other SeNPs-containing bio-nanocomposites. For instance, ROS production in H_2_O_2_-induced HepG2 cells was also significantly decreased in the presence of SeNPs coated with *C. sinensis* EPS [7]. In that study, the inhibition of ROS production seemed to occur in a dose-dependent matter and was more intense as the SeNPs’ size decreased from 150 to 50 nm [7]. In addition, SeNPs capped with *Bacillus paralicheniformis* SR14 EPS were more efficient in reducing the H_2_O_2_-induced ROS production by IPEC-J2 cells than chemically synthesized SeNPs [37], suggesting that the polysaccharide coating might be beneficial for the antioxidant activity.

## 4. Conclusions

The present study showed that the exopolysaccharide produced by the marine bacterium *A. macleodii* Mo169 can be used as a stabilizer and dispersing agent in the formation of SeNPs via the redox system of selenite and ascorbic acid. The synthesized SeNPs had an average particle size of 32 nm and were coated with the polysaccharide, forming a bio-nanocomposite with 297 nm. Thanks to the EPS’s high content in negatively charged functional groups (i.e., carboxylic) and substituents (i.e., pyruvate, sulfate, and lactate), the synthesized SeNPs were stable and well dispersed in the solution. Additionally, the biocompatibility and cellular antioxidant potential of the synthesized EPS/SeNPs bio-nanocomposite was investigated. The SeNPs coated with the EPS had a low cytotoxicity towards CCD-1079Sk fibroblasts and HaCaT keratinocytes, since with concentrations up to 250 mg L^−1^ (containing 23 mg_Se_ L^−1^), high cell viability was maintained. Moreover, the bio-nanocomposite exhibited cellular antioxidant capacity in vitro for concentrations above 125 mg L^−1^ (11 mg_Se_ L^−1^). These results suggest that the EPS was suitable for the stabilization of SeNPs and can have a future in the development of novel biomaterials or formulations with therapeutic applications. In this study, the potential of marine biopolymers in nanotechnology and biomedical applications is also evidenced.

## Figures and Tables

**Table 1:** Particle size of the SeNPs (determined by TEM analysis) prepared with different polysaccharides and the respective polysaccharide/SeNPs bio-nanocomposites (determined by DLS) (PS, polysaccharide; n.a., not available).

Polysaccharide	Size (nm)		Reference
	TEM	DLS	
*Alteromonas macleodii* Mo 169 EPS	22–76/32	297	This study
*Cordyceps sinensis* Cs-HK1 EPS	50	n.a.	[8]
*Gracilaria lemaneiformis* PS	83.6	93–138	[4]
*Grateloupia livida* PS	100	115	[10]
*Larix principis-rupprechtii* PS	n.a.	94–173	[9]
*Lycium barbarum* PS	n.a.	105	[25]
*Oudemansiella raphanipies* PS	60	n.a.	[6]
Carboxylated curdlan	56–65	118–243	[17]
Gum Arabic	34.9	145–170	[24]
Lectinan	33–52	100	[23]
Pectin	41	n.a.	[3]

## Data Availability

Data will be provided upon request.
